# Australian News

**Published:** 1920-04-17

**Authors:** 


### AUSTRALIAN NEWS.

**From Our Special Correspondent.**

### Soldiers and Hospital Appointments.

At a meeting of the Melbourne Hospital Committee held on February 10, the question of giving preference to soldiers in making hospital appointments was again discussed. The subject was brought up by the reading of a letter from the Secretary of the Victoria Branch of the British Medical Association, in which the Council of the Association declared that this preference should be given. Subsequently the Committee received a deputation representing the Returned Medical Officers' Association, the Returned Soldiers and Sailors' Imperial League, and the Sailors and Soldiers' Fathers' Association. After a lengthy discussion, the Hospital Committee resolved to give no pledge with regard to future appointments.

### The Edward Wilson Charitable Fund.

A recent appeal made by the Women's Hospital, Melbourne, for £2,000 worth of radium for treatment of its cancer patients, has been crowned with immediate success. After the public had subscribed about £400, the trustees of the Edward Wilson Charitable Fund have given the whole £2,000 in one sum. This charitable trust is one of the wealthiest in Australia, and it was almost entirely through its generous benefactions that the Melbourne Hospital has been entirely rebuilt on modern lines.

### The Sequel to the Doctors' Strike.

It is stated in Australian newspapers that the friendly societies have been successful in engaging medical officers in England, and the societies reported at a recent meeting that "a number of doctors will shortly be leaving England under contract." By this means it is hoped to overcome the hostility of the Australian Medical Association. What has the British Medical Association to say upon this importation of English doctors?

### The New Health Act.

After having been before Parliament and people for practically the whole of the year 1919, a new Health Act for the State of Victoria has been passed. Having become law, it will wholly supersede the thirty-years-old, much abused Board of Health, which had no power to enforce its findings and rulings. All powers are to be vested in a Commission of seven, including the Chief Health Officer, two other medical practitioners, one member representing cities, towns, and boroughs other than metropolitan municipalities, one member representing shires other than metropolitan municipalities, and a sanitary engineer unconnected with Government, shires, or municipalities. It appears to be a fairly open secret that Dr. Robertson, Chairman of the late Board of Health, who is part author of the new Act, will be chairman of the Commission. The Act has 368 clauses. It will not come into force until a proclamation has been issued by the State Executive, and so many preliminary details still have to be attended to that the proclamation was not expected before March. But the Ministry intended to appoint at once the seven members of the Health Commission. The Commission's first work will doubtless be the appointment of district health officers. As the B.M.A. and the Friendly Societies are not yet reconciled, it is indeed likely that the Commission will have to appoint a second set of medical officers to look after school children and the sick poor.

